# Forecasting electric vehicles sales with univariate and multivariate time series models: The case of China

**DOI:** 10.1371/journal.pone.0176729

**Published:** 2017-05-01

**Authors:** Yong Zhang, Miner Zhong, Nana Geng, Yunjian Jiang

**Affiliations:** School of Transportation, Southeast University, Jiangsu, Nanjing, China; Chongqing University, CHINA

## Abstract

The market demand for electric vehicles (EVs) has increased in recent years. Suitable models are necessary to understand and forecast EV sales. This study presents a singular spectrum analysis (SSA) as a univariate time-series model and vector autoregressive model (VAR) as a multivariate model. Empirical results suggest that SSA satisfactorily indicates the evolving trend and provides reasonable results. The VAR model, which comprised exogenous parameters related to the market on a monthly basis, can significantly improve the prediction accuracy. The EV sales in China, which are categorized into battery and plug-in EVs, are predicted in both short term (up to December 2017) and long term (up to 2020), as statistical proofs of the growth of the Chinese EV industry.

## Introduction

In China, vehicle emissions have been increased rapidly recent years. In 2014, China’s crude oil import dependency reached 59.6%, the future economic development would be restricted by the inadequate energy supply of traditional fossil fuel [1]. At the same time, China’s CO2 emissions was growing fast and reached 9.5243 billion tons, accounting for 27.1% of the world’s CO2 emissions in 2013 which among them vehicle emissions accounted for 15.9% of total carbon dioxide emissions [2]. The transition toward a substantially carbon and energy-efficient transport system is an ongoing global process. The electrification of the transportation sector through the diffusion of electric vehicles (EVs) is considered a promising pathway to reduce air pollution from on-road vehicles and to strengthen energy security. As a statistical standard in China, EV sales are recorded in two categories, namely, battery EVs (BEVs) and plug-in EVs (PHEVs). BEVs derive all power from battery packs while PHEVs derive some power from electricity and some from gasoline. Both usages lead to positive environmental effects and the improvement of urban health [3], for example, plug-in hybrid electric vehicle (PHEV) technology offers a possible approach to reducing and dependency on oil as a transportation fuel [4]. While the adoption of EVs is affected by many barriers, such as high retail prices, the limited range of batteries, and the lack of supporting infrastructure, particularly charging stations [5]. Thus, forecasts of market demand are divided into BEV and PHEV sales amounts, and the overall performance can be drawn by combining the results.

**Fig 1:**
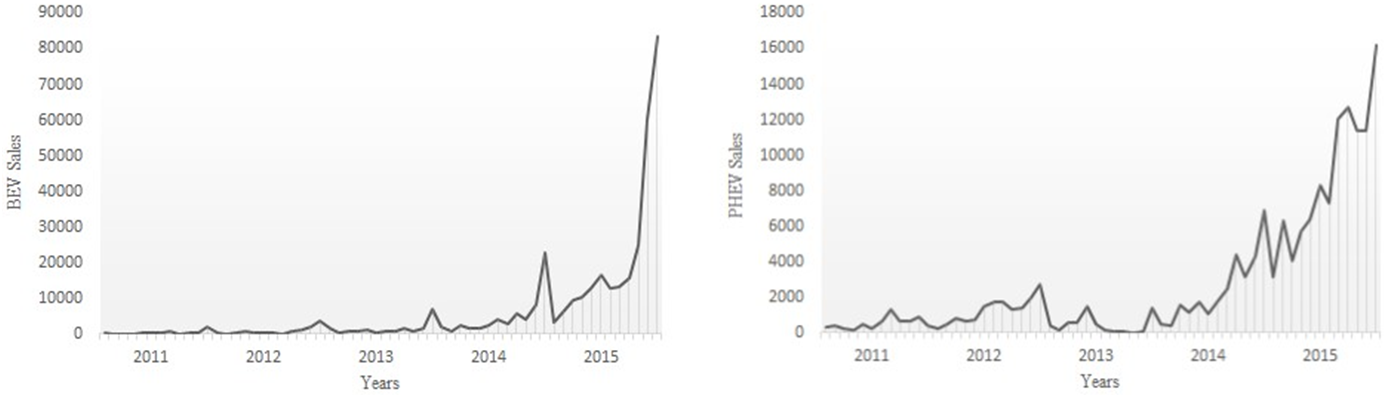
Sales of BEV and PHEV in China from January 2011 to December 2015.

Dependencies on diverse macroeconomic environment also hinder the accurate long-term prediction of the future of automotive sales [6]. Thus, the market diffusion of EVs has had mixed results thus far. A prediction of nearly 3.3 million electric cars would be sold in the US, with approximately 500,000 sold in the west coast in 2020 [7]. A forecast of the sales in the US regarding different EVs is made in similar ways (i.e., 16 million for BEV and 13 million for PHEV) [8]. The main time series considered in this study is the sequence of the monthly EV sales in China from January 2011 to December 2015, as presented in statistical reports [9]. The data set comprises the EV sales in two segments (i.e., BEV and PHEV). Although BEV and PHEV appear to have different structures that control the underlying dynamics in the short term, a similarity in long-term trends can be observed (as shown in Fig 1).

Forecasting sales and demand over a monthly horizon is crucial for planning the production processes of automotive and other complex product industries [6]. An improved prediction is often assumed to be obtained with a multivariate time series than by a scalar time series. However, the multivariate time series can hardly extract component information that is beneficial for the prediction process. From this point of view, univariate prediction may occasionally be superior to multivariate prediction [10]. Scholars all over the world have been developing new methods to achieve accurate prediction respectively from the univariate time-series and the multivariate time series. On the one hand, singular spectrum analysis (SSA) which was developed to model univariate time series [11] is a relatively new nonparametric and data-driven technique [12]. Recent empirical studies that apply the SSA methods on economic and financial data have shown satisfactory results. On the other hand, the vector autoregressive model (VAR), as a structural relationship identification methodology for multivariate time series [13], can identify the dynamic couplings between EV sales and economic indicators.

Therefore, the current study proposes models to forecast the quantified market demand for EV. First, an SSA model is applied to build univariate time-series forecasting models. Second, a multivariate VAR is built by considering the external influences from six economic indicators. Finally, comparisons of the models are conducted, and the EV sales are predicted in the short and long terms.

## Literature review

### Electric vehicle market demand

There are three scenarios of PHEV market penetration in the US (namely, hybrid technology-based assessment, R&D goal achievement, and supply constraint), and the penetration rate is predictable [14]. One recent market assessment conducted by EPRI and NRDC forecast PHEV penetration rates of 62% by 2050 [15]. The penetration can be described with spatially explicit consumer choice model, thereby showing that it could be enhanced by the readily available estimates of the expected lifetime fuel costs, as well as increases in gasoline prices and synergetic gasoline tax [16]. Besides, the sales and range anxiety of EVs could be addressed through parking layout, policy, and regulation [17]. And the market potential of PHEVs for the German commercial passenger car sector in 2020 [18]. Some other researches focus on demonstrating how policy entrepreneurs influence and accelerate the introduction of PHEVs in local public authorities [19].

There have been many efforts evaluating the potential of PHEVs from the supply side. To optimize the energy management of hybrid electric vehicles, velocity prediction models were built based on exponentially varying, Markov-chain, and Neural Network respectively. Potential of the each models are analysed with the prediction precision, computational cost, and fuel economy. The main conclusion is that the NN-based models show the best overall performance [20]. Some other studies focus on key strategies to minimize carbon emissions of PHEVs. A series of influential factors were discussed from the angle of both the vehicles and the grids. And different scenarios were built to describe real-life cases and quantify carbon reduction of each scenario [21]. Also, cost-optimal control model was built to maintain the best economy during charging and operation [22].

From the demand side, relatively fewer studies were conducted to describe the public acceptance and preference for EVs. In US cities, consumers’ preference for PHEVs was examined with data collected from online surveys in US cities [23]. Similarly, in Malaysia, the intention of consumers to use electric vehicles was described with survey-based data collected from questionnaires [24]. The regression model shows that consumer’s intention are strongly related to a lot of influential factors, including social, financial, environmental aspects. In recent years, a more comprehensive indicator was introduced using data displayed in Google Trends [25]. Its performance in forecasting private consumption show that the new indicator derived from Google shows batter performance in comparison to traditional survey-based indicators. There are also some studies utilizing this indicator to describe the online browsing habits and indicate consumer preferences of automobile market [26]. The results are that this indicator not only fits the forecasting model well, but also helps to locate the turning point in sales trends. Similar conclusions were made in the experiments done by some other researchers, showing that searching data in Google Trends have strong predictive value [27]. Baidu Index from Baidu Search Engine in China is a dependable source of historical searching data similar to Google Trends. Therefore, this paper considers the results of previous scholars, focusing on the consumer perspective to research electric vehicle market demand.

### Vehicle sale forecast

Automobile sales forecasting has received significant attention. Most studies have focused on time-series models. Predictions of the Chinese automobile sales can be made with support vector regression [28] and methods alike. Moreover, researches find that by applying data mining algorithms to model the automobile markets, the economic indicators are proved to be relate to sales [29]. Economic indicators related to automobile market include gross domestic product, consumer price index (CPI), interest rate, unemployment rate, and gas prices with automobile sales [30]. To address the relationship among these variables, an adaptive network-based fuzzy inference system can be made to estimate new automobile sales [31]. Other indicators such as subsidies, range, charge point availability, emission rate, and revenue tax are also used for forecasting the future demand for EV [32]. As shown in Table 1, forecast results related to sales of EV differ greatly by researchers, times, as well as countries. Results by different models predict are not the same, but we can see obvious rising trends can be found in all predictions. Forecasting of EVs market is complicated by economics, finance, systems engineering and all kinds of factors, it is difficult to model using the classical tools of market forecasting. In this paper, we predict the EVs sale from univariate and multivariate methods respectively, and give the most reasonable prediction method and prediction conclusion through comparison.

**Table 1:** Forecast results in different countries.

Country	Topic	Forecast results
U.S.	A forecasts model for EV. [33]	EV sales of 3.2m (0.5m in west coast and 2.8m in the rest) in 2020
Istanbul	EV diffusion scenarios. [34]	Market share of BEV and HEV would reach 19.76% and 20.77% respectively by 2042
U.K.	Factors affecting EV demand. [32]	Sales of 0.538 million for BEV and 2.4 million for PIHV in 2030
Boston (U.S.)	EV diffusion with logit model. [35]	EV as a percentages of vehicle stock is predicted up to 22% by 2030
U.S.	EV sales projections. [36]	EV sales are predicted to be from 1.8 to 7.3 million vehicles by 2023
Portugal	EV diffusion. [37]	EVs can reach 7.6% of vehicle fleet, hybrid vehicles reach 60% by 2030

### Methodologies

Multivariate time-series models may be expected to generate accurate forecasts. However, the univariate forecasting models may considerably outperform the multivariate models in certain conditions [38], such as scenarios when the prediction steps were small [10]. However, some researchers think that the univariate models performed similarly to the multivariate models [39]. Additional comparisons of the two models can be determined in different fields, such as the prediction of emergency department demand [40] and energy market volatility [41].

SSA is a univariate time-series model and does not rely on a priori defined functions; however, it generates a set of components directly from the time-series under study [42]. Unlike in traditional time-series models, the trend is any gradually varying component of the series that does not contain cyclical or seasonal components [11]. The SSA model is also nonparametric; hence, it is not limited by linearity, normality, and stationarity of the data. Applying SSA to measure the nonlinear dependency of financial markets shows more accurate than conventional univariate time-series models [43].

VAR is a multivariate time-series model and is a combination of the autoregressive method and the Box–Jenkins method developed by George Box and Gwilym Jenkins in 1976 [44]. The VAR model has been extensively applied in different fields, such as the sales prediction of real estate [45], automobile market share [46], economic indicators [47], and rainfall patterns [48].

In summary, this study uses previous research on automobile sales prediction as basis to apply the SSA and VAR models to forecast the market demand of EVs by considering the characteristics of the EV market penetration.

## Time series models for sales forecast

### SSA model

#### Methodology

The main objective of SSA is to decompose the original series into a sum of series; thus, each component can be identified as a trend, periodic, quasi-periodic, or noise[49]. Four steps are conducted, namely transformation, decomposition, grouping, and reconstruction [50].

Step 1: Transformation. Embed the sampled time series in a vector space of dimension M*N and the trajectory matrix X.

$$\text{X}={\left(x_{ij}\right)}_{i,j=1}^{k,}=\left[\begin{array}{cccc} x_{1} & x_{2} & \ldots & x_{M}\\ x_{2} & x_{3} & \ldots & x_{M+1}\\ \ldots & \ldots & \ldots & \ldots \\ x_{k} & x_{k+1} & \ldots & x_{N} \end{array}\right]$$ (1)

Step 2: Decomposition. Compute the M*M lag-covariance diagonal matrix C_D_. The components of X are uncorrelated because E comprises the orthogonal vector E_K_. The diagonal matrix Λ comprises the ordered values 0 ≤ *λ*_1_ ≤ *λ*_2_…≤ *λ*_*M*_ with square roots that are the singular values.

$$C_{D}=\frac{1}{N-M+1}\overset{N-m+1}{\underset{t=1}{\sum }}x_{i+t-1}x_{j+t-1}$$ (2)

$$C_{D}=E\Lambda E^{T}$$ (3)

$${\left(XE\right)}^{T}\left(XE\right)=\Lambda$$ (4)

Step 3: Grouping. Recovering the original time series, and the principal components are convolved with the following associated eigenvectors.

$$a_{i}^{k}=\overset{m}{\underset{j=1}{\sum }}x_{i+j-1}e_{j}^{k},(i=1,2,\ldots ,N;j=1,2,\ldots ,M;k=1,2,\ldots ,M)$$ (5)

$$x_{i+j-1}=\overset{m}{\underset{k=1}{\sum }}a_{i}^{k}e_{j}^{k}$$ (6)

Step 4: Reconstruction. An initial guess $\hat{x}(0)$ of the prediction is added, and repeated the procedure. From the augmented time series with *N*_*t*+1_ values, the prediction value is updated with $\hat{x}(1)$ by replacing $\hat{x}(0)$.

$$\hat{x}(1)=x_{N_{t}+1}^{'}=\sum_{k=1}^{d_{s}}a_{N}^{k}e_{m}^{k}$$ (7)

Where $a_{N}^{k}$ is the value of principal component and $e_{m}^{k}$ is the component of eigenvector in the augmented trajectory matrix X. The process is iterated until $\hat{x}(q)$ is accepted as the predicted value for $\hat{x}(N_{t}-1)$.

$$\left|\hat{x}(q)-\hat{x}(q-1)\right|<\varepsilon ,(q=1,2,\ldots .)$$ (8)

#### Empirical test

**Fig 2:**
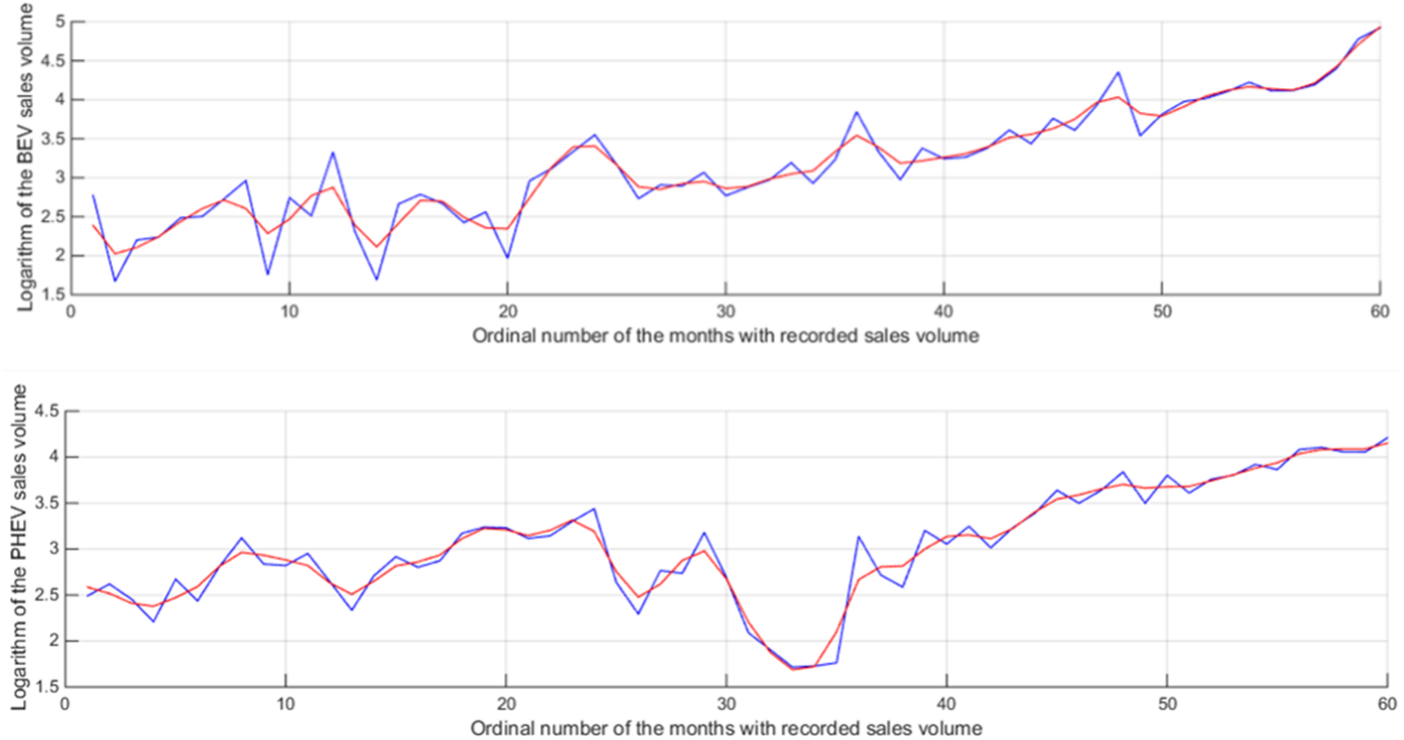
Fitness of the BEV and PHEV sales values with the SSA model.

We scale each data series according to the logarithm to ensure that all series have the same scale. To obtain accurate forecast results, the window length for the BEV sales time series is defined as 8 and that for PHEV sales is 9. The number of components is defined based on the contribution of each component to the variance of the time series. In this case, four and five components are selected for the BEV and PHEV time series, respectively. Accordingly, the sum of their contributions is at least a predefined threshold (> 90%). Fig 2 shows the forecast results. The trends of the BEV and PHEV sales are obtained satisfactorily, although a relatively large error is determined with regard to the monthly data.

### VAR model

#### Methodology

First, the possible existence of unit roots is analysed to ensure that the model is stationary in terms of the variables used [51]. If a time series has a unit root, then the difference of the series is conducted until it is stationary. Subsequently, co-integration tests are conducted, and an error-correction term should be added. Ganger causality tests further explain the relationships [52]. Finally, the VAR model is developed and it forecasts with the recursive method.

The time series is expressed as follows, where y_t_ is the current value, p is the lag in the autoregressive process, with y_*t*−1_,…,*y*_*t*−*p*_ means the measurement values, and *β*_0_,…,*β*_*p*_ means the regression coefficient.

$$y_{t}=\beta_{0}+\beta_{1}y_{t-1}+\beta_{2}y_{t-2}+\ldots +\beta_{p-1}y_{t-p+1}+\beta_{p}y_{t-p}+\varepsilon \varepsilon_{t}$$ (9)

Step 1: Unit root test. The stationarity, in which the mean and variance are constant and the covariance is not time dependent, is tested using the augmented Dickey–Fuller method [53]. Where △*y*_*t*_ is the time series value of 1 − t time periods, *θ* is the sum of constant values, and *ϕ*_*i*_ is the trend coefficient. The probability of the hypothesis that unit roots exist is tested with *H*_1_:*θ*<0. If the data are not stationary, then the differencing process should be conducted.

$$△y_{t}=\beta_{0}+\theta y_{t-1}+\sum_{i=2}^{p}\phi_{i}△y_{t-1+i}+\varepsilon \varepsilon_{t}$$ (10)

Step 2: Co-integration test. To verify the existence of co-integration between *x*_*t*_ and *y*_*t*_, a regression is run to assess if the residual *z*_*t*_ is stationary. If *z*_*t*_ is stationary, then *x*_*t*_ is co-integrated with *y*_*t*_. Thus, the first-order difference of each series, lagged regression residual, and error correction should be included in the modelling procedure [54].

$$y_{t}=\alpha +\beta x_{t}+z_{t}$$ (11)

Step 3: Granger causality test. The causality test is conducted using an F-test on the coefficients of the *x* lagged values in the regression of *y*.

$$△y_{t}=c_{1}+\sum_{i=1}^{m}\beta_{1i}△y_{t-i}+\sum_{i=1}^{n}\delta_{1i}△x_{t-i}+\gamma_{1}u_{1t-1}+\varepsilon_{1t}$$ (12)

$$△x_{t}=c_{2}+\sum_{i=1}^{p}\beta_{2i}△x_{t-i}+\sum_{i=1}^{q}\delta_{2i}△y_{t-i}+\gamma_{2}u_{2t-1}+\varepsilon_{2t}$$ (13)

Time series △*y*_*t*_ and △*x*_*t*_ are assumed, where *u*_1*t*−1_ and *u*_2*t*−1_ are the error-correction terms; *ε*_1*t*_ and *ε*_2*t*_ are the residuals of the model; and *m*, *n*, and *p* are the numbers of lags. It is estimated using the ordinary least square method, supposing as a seemingly unrelated regression equation [31].

$$FPE(m)=(\frac{T+k}{T-k})(\frac{SSR(m)}{T})$$ (14)

Where *T* is the sample size, and *k* is equal to *m* + 1 if the two series are co-integrated and *m* + 2 if not. By fixing *m* at its optimal value m^*^ and varying *n* to minimize *FPE*(m^*^,*n*), if *FPE*(m^*^,n^*^)<*FPE*(m^*^), then *x* granger causes *y*.

Step 4: Establishing models. The lag value is determined by calculating Aikake information criterion (AIC).

$$AIC=\log\sigma_{k}^{2}+\frac{n+2k}{n}$$ (15)

$$\sigma_{k}^{2}=\frac{\sum_{i=1}^{n}{\left(y_{i}-y_{r}\right)}^{2}}{n}$$ (16)

Where *y*_*i*_ is the observed value, and *y*_*r*_ is the mean. The lag value is better taken when AIC is small [38]. The model comprising the time series of the EV sales and all other variables established as follows. Where *c* is the constant indicating the intercept and *ε* is the error level.

$$\left[\begin{array}{c} y_{1,t}\\ y_{2,t}\\ \ldots \\ y_{7,t} \end{array}\right]=\left[\begin{array}{c} c_{1}\\ c_{2}\\ c_{7} \end{array}\right]+\left[\begin{array}{cccc} a_{1,1}^{1} & a_{1,2}^{1} & \ldots & a_{1,7}^{1}\\ a_{2,1}^{1} & a_{2,2}^{1} & \ldots & a_{2,7}^{1}\\ \ldots & \ldots & \ldots & \ldots \\ a_{7,1}^{1} & a_{7,2}^{1} & \ldots & a_{7,7}^{1} \end{array}\right]\left[\begin{array}{c} y_{1,t-1}\\ y_{2,t-1}\\ \ldots \\ y_{7,t-1} \end{array}\right]+\ldots +\left[\begin{array}{cccc} a_{1,1}^{p} & a_{1,2}^{p} & \ldots & a_{1,7}^{p}\\ a_{2,1}^{p} & a_{2,2}^{p} & \ldots & a_{2,7}^{p}\\ \ldots & \ldots & \ldots & \ldots \\ a_{7,1}^{p} & a_{7,2}^{p} & \ldots & a_{7,7}^{p} \end{array}\right]\left[\begin{array}{c} y_{1,t-p}\\ y_{2,t-p}\\ \ldots \\ y_{7,t-p} \end{array}\right]+\left[\begin{array}{c} \varepsilon \varepsilon_{1,t}\\ \varepsilon \varepsilon_{2,t}\\ \ldots \\ \varepsilon \varepsilon_{7,t} \end{array}\right]$$ (17)

Step 5: Predicting. First estimate the equations and obtain all coefficients with a set of history observations. Thereafter, one-month-ahead values are forecasted. As time advances, we re-estimate the equations with the updated data and obtain the out-of-sample forecast.

#### Empirical test

**Fig 3:**
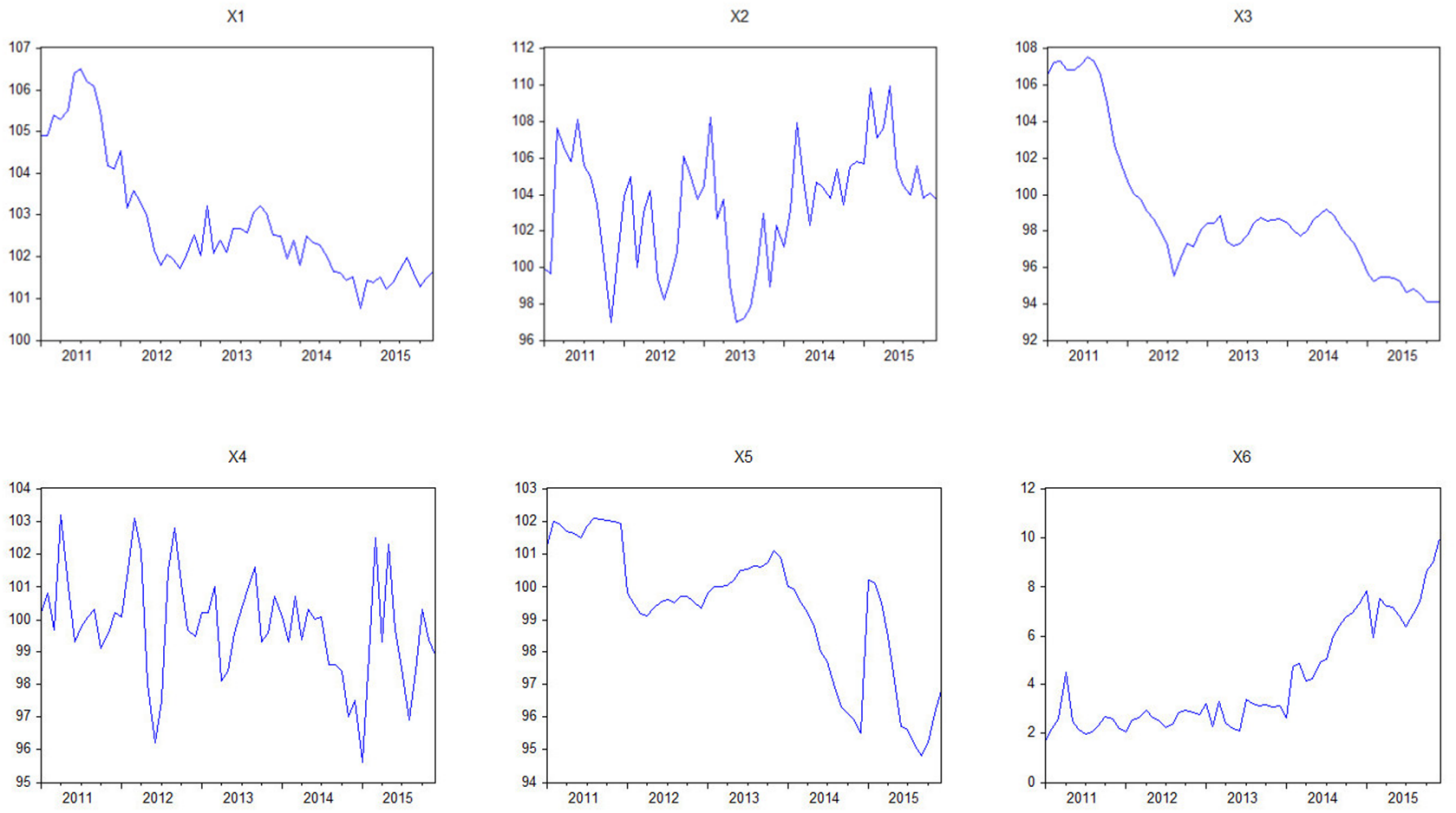
Economic indicators from January 2011 to December 2015.

The selection of economic indicators, which is intended to improve the forecast of the BEV and PHEV sales, should be able to reveal a structural relationship between the EV sales and macroeconomic environment. By considering the modelling restrictions (i.e., multi co-linearity, redundancy, and over-parameterization), six monthly indicators are selected, and the following notations are introduced for ease of presentation. The historical sales of BEV and PHEV (Y_1_ and Y_2_) were collected from Energy-saving and New Energy Vehicle Yearbook [9]. The economic indicators (X_1_, X_2_, X_3_, X_4_, and X_5_) were collected from China Statistical Yearbook [55], which can be downloaded freely from the website of National Bureau of Statistics of China. Baidu Index from Baidu Search Engine in China is a dependable source of historical searching data. The new indicator “Baidu data” (X_6_) was collected from Baidu Search Engine by searching the keyword “electric vehicle”. Specifically, to test the feasibility of using this new indicator to show Chinese consumer’s preference towards purchasing EVs, we conducted correlation analysis between EV sales (Y_1_ and Y_2_) and the Baidu data (X_6_). The Pearson correlation coefficients is 0.71 and 0.84, and the T-statistics is 7.62 and 11.82 respectively. Therefore it can be concluded that both Y_1_ and Y_2_ shows strong correlation with X_6._ All data are available in S1 and S2 Tables in the Supporting Information. We calculated and normalized the value of each indicator. As showed in Fig 3, most of the selected indicators show nonlinear patterns.

Y_1_: Number of BEV sales on a monthly basis;
Y_2_: Number of PHEV sales on a monthly basis;
X_1_: Consumer price index, measuring the changes in the price of a market basket of consumer goods purchased by households
X_2_: Consumer confidence index, measuring the optimism that consumers are expressing through savings and spending activities
X_3_: Producer price index, measuring the average changes in prices received by domestic producers for their output
X_4_: Fuel retail price, the average price of fuels on a monthly basis
X_5_: Vehicle price, the average price of all automobiles on a monthly basis
X_6_: Baidu data, indicating the searching frequency of particular terms about consumers’ interest on EVs [56].

We scale the time series of all eight variables according to the logarithm to ensure that they have the same scale. The result of the unit root tests shows that log (y_1_), log (y_2_), log (x_2_) and log (x_4_) are stationary, whereas other variables are not. However, for log (x_1_), log (x_3_), log (x_5_), and log (x_6_), the plots of the first-order difference indicate stationary behaviour.

Regression among time series is conducted, and the results show that no co-integration exists among the eight time series, and predictions can be made without exogenous variables. The Granger causality test is used to determine the causality relationships between the BEV and PHEV sales and indicators.

**Fig 4:**
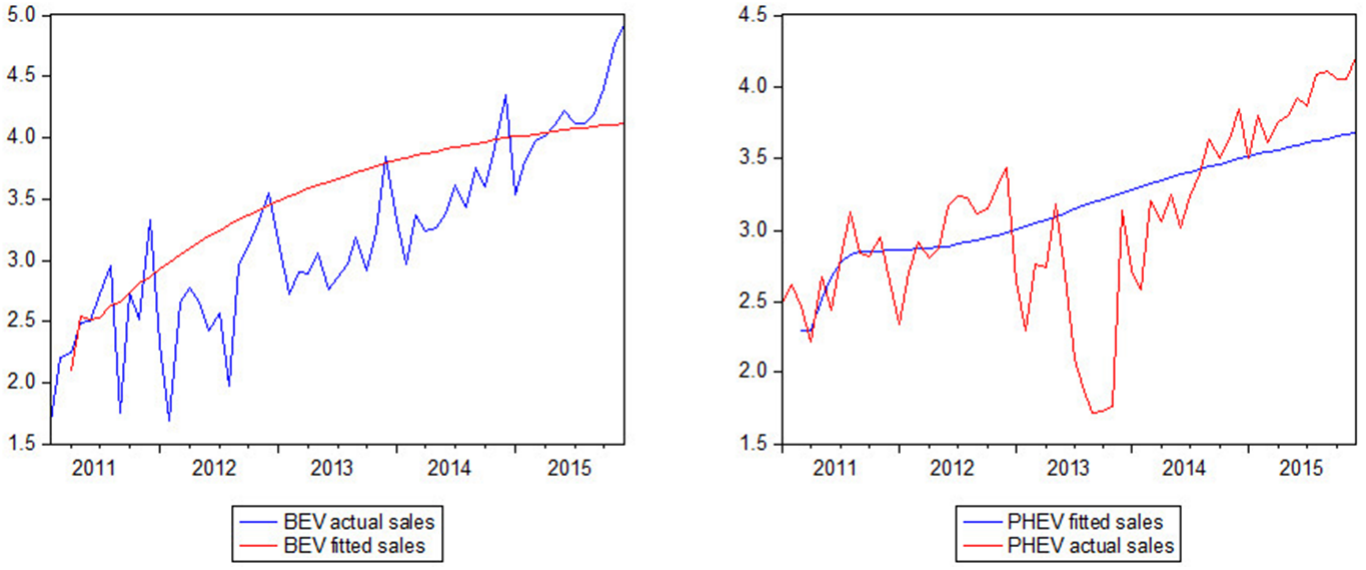
Fitness of the BEV and PHEV sales values with the VAR model.

The VAR model with error correction is constructed. The coefficients of the equation are obtained with observations to obtain the forecast result (Fig 4). The result shows that the sales prediction is reasonable to be based on these indicators. In the out-of-sample forecast, we use ARIMA to obtain the predicted value of input parameters. As the main objective of this paper is to build and discuss two sales-forecast models for EVs, we will not cover the ARIMA model in this paper.

## Results and discussions

### Model performance

Given that the SSA and VAR models are obtained and able to be used for forecasting, the forecast accuracy level can be evaluated mathematically with Mean absolute percentage error (MAPE) and Normalized root-mean-square error (RMSE).

MAPE is a computation of mean absolute error to show how close the forecasted and real values are. Where *F*_*t*_ is the forecast value, *Y*_t_ is the actual value, and n is the length of time series.

$$MAPE=\frac{1}{n}\sum_{t=1}^{n}\left|\frac{Y_{t}-F_{t}}{Y_{t}}\right|$$ (18)

RMSE is the root-mean-square error, where ${\hat{y}}_{T+i}$ is the one-step ahead forecast value of *y*_*T*+*i*_. If either RMSE = 0 or RMSE is small, then the prediction is perfect or varies accurately

$$RMSE=\frac{{\left[\sum_{i=1}^{N}{\left({\hat{y}}_{T+i}-y_{T+i}\right)}^{2}\right]}^{1/2}}{{\left[\sum_{i=1}^{N}{\left({\hat{y}}_{T+i}-y_{T+i-1}\right)}^{2}\right]}^{1/2}}$$ (19)

In this case, the observations of the EV sales from January 2011 to December 2014 are used as sampled data for model building, and the 12-step-ahead forecasts are conducted. The predicted values are compared with the actual observations from January 2015 to December 2015. For prediction models, one of the main performance measurement is precision. The SSA model indicates satisfactory results, that is, 36.40% in MAPE and 21.5% in RMSE. However, the VAR model presents substantially accurate forecasting results in terms of MAPE and RMSE, with average values of 29.1% and 16.31%, respectively. Comparing the two models from the perspective of data collection and handling, we found that SSA have lower data requirements. SSA is a kind of principal component analysis, with strong noise reduction ability. The VAR model can be used to obtain a more precise prediction. As both economic factors and consumer preferences are considered, the forecasted results have better interpretation. However it should be noted that the value of economic factors used in VAR models are mostly collected from the Statistics Yearbooks. Thus there will always be a lag period. In summary, for emerging market like EV market in China and some other country, both univariate and multivariate forecast models yield reasonable results. SSA is a satisfactory model with less data requirements, and VAR is a traditional model that can consider both economic factors and consumers’ preference.

### Short-term sales forecast

**Fig 5:**
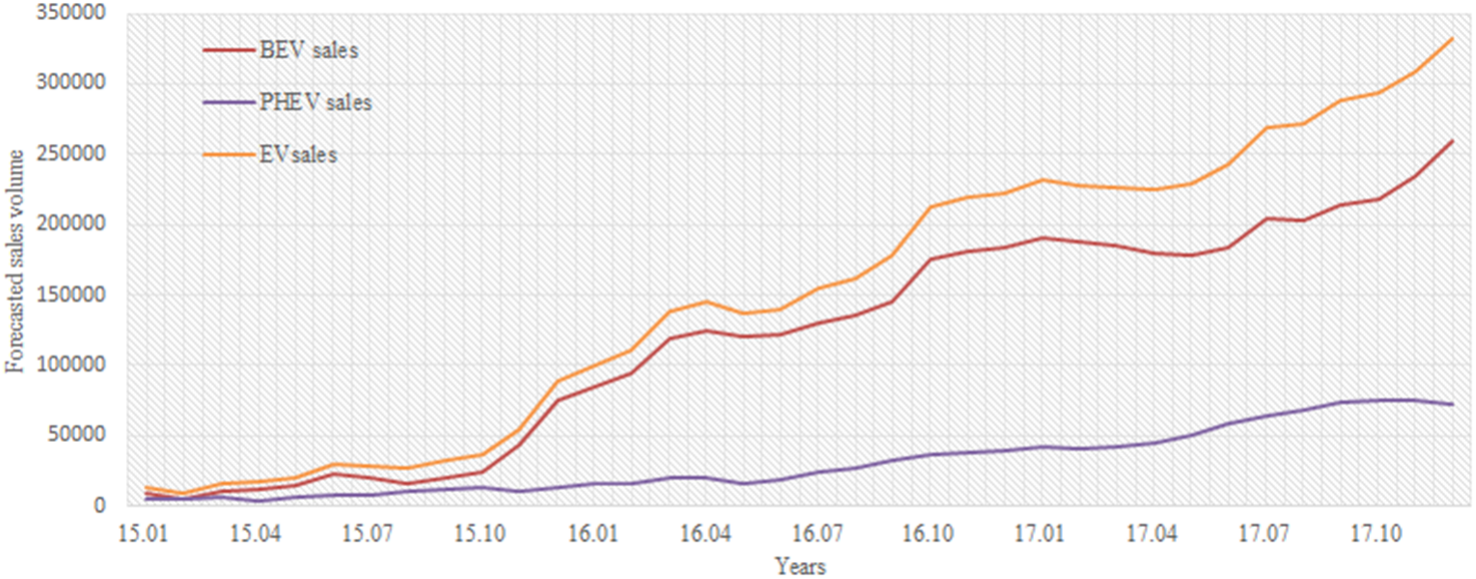
Short-term forecast of EV sales up to December 2017.

Fig 5 shows that the sales of BEV and PHEV from January 2016 to December 2017 are predicted. Both time series indicate an apparent rising trend. For the BEV markets, the growth trend continues to December 2017, followed by slight fluctuations in the late 2016 and the early 2017. At the end of 2017, a total of 258394 BEVs would be sold. For the PHEV markets, the growth of sales is relatively smooth and finally reaches a total of 72957 sold PHEVs.

### Long-term sales forecast

**Fig 6:**
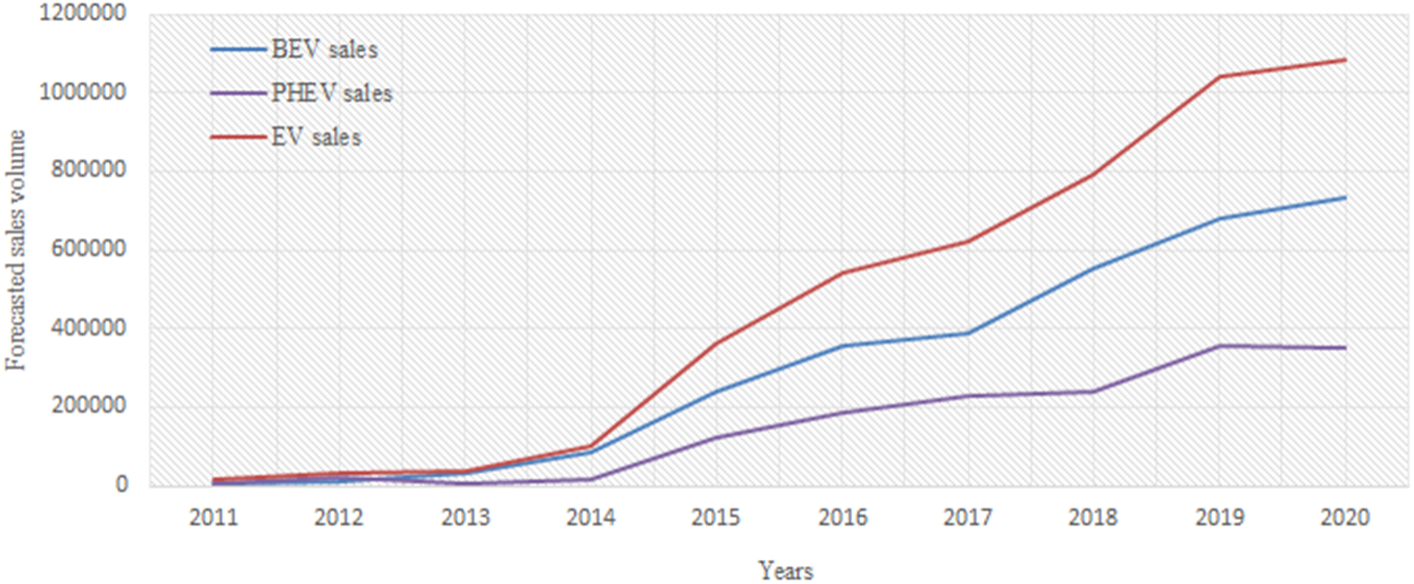
Long-term forecast of EV sales up to 2020.

The long-term trends of the EV sales are also predicted using the VAR model, with the sum of the sales for 12 months indicating the sales of the year (see Fig 6). A relatively accurate understanding of the future EV market penetration in China is indicated. The BEV and PHEV markets will be presenting a substantial increment in demand in the next five years. In particular, nearly 0.35 million BEVs and 0.72 million PHEVs would be sold in 2020.

Corresponding to the strategic plan made by the government, EVs will somehow overhaul the Chinese automobile markets in the future. However, although the predicted results may seems optimistic, the models yield a reasonable prediction of the growing trend (similar conditions can be found in the studies in [32]. The predictions made by the mathematical models on solid foundation and both results (either from official documents or from mathematical models) are of homogeneous significance to the EV market.

## Discussions

Penetrations of EV can lower health-impairing pollutants and greenhouse emissions, thereby providing sources of domestic employment and investment. Sales forecast plays a prominent role in business strategy to propel penetration and generate revenues. A univariate time-series model, which depicts the accurate tendency of EV sales, can be applied easily because only a few observations are required. A multivariate model, which accounts for an improved performance in terms of predicting error, can also be used for reliable monthly and yearly forecasts.

Our results of EV sales show rising trend, corresponding with that in the U.S. and U.K. Yet the numerical result is bigger due to the large vehicle fleets that China already has. The multivariate models result in a better forecasting performance than the univariate ones. Exogenous parameters that influence the sales market of the Chinese EV industry, including consumer price and confidence, producer and retailer prices, as well as the influence from fuel markets, are good predictors of the Chinese EV sales according to causality tests. For market participants who concentrate on generating revenue, they need to focus on the variation tendency of the market demand while analysing the economic indicators. For policy makers, incentive strategies can be formulated based on the multivariate models, such as subsidies, tax adjustment, and employment encouragements. Implications to manufacturers and researchers also include the understanding of the dynamic relationship between a single market (i.e., BEV or PHEV market) and the entire EV market.

### Suggested policy

For the Chinese market, positive penetrations are predicted. Thus, incentive policies can be implemented. The affordability of EV, even a quintupling of battery size at no additional costs improves EV adoption by 5% [57]. Thus, improvements made to reduce full social lifetime cost is a direct incentive for the boost of EV market, including the size and lifetime of key components (i.e., batteries, electric motors), the cost of key materials (i.e., lithium, platinum and membrane) and the maintenance and repair requirements.

Diffusion of EV market is greatly influenced by customer perception and awareness. Thus, propagating the high-quality, vast-classifications as well as the great-sustainability signals will benefit the sales of EV. Besides, the penetration is directly related to travel demand, which can be estimated with economic indicators.

Usually, incentive policies include the subsidy-based and the tax-based policies. The former include purchase, charging as well as maintenance subsidies. The latter are demonstrated in tax on vehicle purchase, circulation as well as electricity cost. In the case of China, effects of policy may also be manifested directly in the vehicle fleet (PHEV bus production in China has increased dramatically from 2010 to 2013 [58]).

Enhancing the recharging infrastructure, and the availability of fast-charging points is another direct incentive. A potential buyer will not purchase an EV unless he/she is assured of having constantly-available charging places. Within the charging stations, the parking configurations, charger design as well as legislation contribute to its efficiency [17].

Other incentives include urban sprawl control and lane access that are specially designed for EV; free parking or electricity; exemption of emissions test; and better insurance products. Forecasting results are significant to the expanding EV industry in China. On the one hand, short-term forecasts enhance the understanding of the changing EV market and provide a dependable foundation for market participants, such as producers, retailers, and consumers. For the participants in the Chinese EV market, corresponding strategies should be implanted to solve problems that will arise from the expanding production, inventory, and transportation. On the other hand, long-term forecasts are significant for the EV industry because of the lengthy period of time required for production processes.

## Conclusions

In this study, an SSA model is built as a univariate time-series model and a VAR as a multivariate one. Both models are applied to forecast the sales of BEV and PHEV in China with 60-month observations from January 2011 to December 2015. The results show that the VAR model is considerably suitable, which considers the effect of economic indicators, including consumer price, consumer confidence, producer price, fuel and vehicle price and Baidu data (indicator that measures the tendency of the curiosity and interest of consumers on the EV market).

This study contributes to the forecast model in three aspects, namely, the modelling framework, comparison of models, and model application. Regarding the modelling framework, interpretable models that provide reasonable forecasts for a relatively niche market, such as the EV market in China, can be created, and monthly collection of data is suitable. Multivariate time-series models may better explain the future EV market than the univariate methods. The EV sales in China are forecasted using the VAR model in the short term (January 2016 to December 2016), thereby indicating rising trends in the BEV and PHEV sales and the monthly sales of 194783 and 38270, respectively. The trends of sales in the next five years are also described. The results confirm a positive future for the BEV and PHEV markets in China, which correspond to what is expected in the strategic plan by the government. Thus, policy makers and members of the EV supply chains are able to implement reasonable regulating, producing, and retailing plans. Nevertheless, our model does not account for the unobserved heterogeneous variables, such as policy and regulation. Future studies on the forecast of EV sales and model selection are expected.

## Supporting information

S1 Table Sales of BEV and PHEV in China from January 2011 to December 2015. (DOCX)

S2 Table Economic indicators from January 2011 to December 2015. (DOCX)
