# Supplementary material for: Forecasting electric vehicles sales with univariate and multivariate time series models: The case of China
Source: PLoS One. 2017 May 1;12(5):e0176729. doi: 10.1371/journal.pone.0176729 (PMC5411096; doi:10.1371/journal.pone.0176729)
Supplement: S1 Table — (DOCX) [file pone.0176729.s001.docx]

**S1 Table. Sales of BEV and PHEV in China from January 2011 to December 2015**

| Year/Month | BEV sales | PHEV sales |
| --- | --- | --- |
| 2011/01 | 588 | 310 |
| 2011/02 | 47 | 417 |
| 2011/03 | 159 | 286 |
| 2011/04 | 173 | 162 |
| 2011/05 | 305 | 470 |
| 2011/06 | 319 | 273 |
| 2011/07 | 535 | 650 |
| 2011/08 | 917 | 1323 |
| 2011/09 | 57 | 688 |
| 2011/10 | 554 | 663 |
| 2011/11 | 325 | 896 |
| 2011/12 | 2137 | 443 |
| 2012/01 | 205 | 216 |
| 2012/02 | 49 | 512 |
| 2012/03 | 461 | 826 |
| 2012/04 | 610 | 635 |
| 2012/05 | 469 | 746 |
| 2012/06 | 266 | 1483 |
| 2012/07 | 364 | 1728 |
| 2012/08 | 93 | 1701 |
| 2012/09 | 910 | 1306 |
| 2012/10 | 1303 | 1395 |
| 2012/11 | 2161 | 1999 |
| 2012/12 | 3553 | 2741 |
| 2013/01 | 1476 | 436 |
| 2013/02 | 540 | 197 |
| 2013/03 | 808 | 583 |
| 2013/04 | 784 | 546 |
| 2013/05 | 1167 | 1517 |
| 2013/06 | 588 | 488 |
| 2013/07 | 752 | 123 |
| 2013/08 | 935 | 80 |
| 2013/09 | 1556 | 52 |
| 2013/10 | 847 | 1 |
| 2013/11 | 1683 | 58 |
| 2013/12 | 6985 | 1373 |
| 2014/01 | 2169 | 523 |
| 2014/02 | 948 | 387 |
| 2014/03 | 2386 | 1589 |
| 2014/04 | 1753 | 1136 |
| 2014/05 | 1837 | 1767 |
| 2014/06 | 2408 | 1037 |
| 2014/07 | 4090 | 1709 |
| 2014/08 | 2731 | 2460 |
| 2014/09 | 5755 | 4358 |
| 2014/10 | 4071 | 3160 |
| 2014/11 | 8475 | 4346 |
| 2014/12 | 22687 | 6897 |
| 2015/01 | 3451 | 3148 |
| 2015/02 | 6519 | 6334 |
| 2015/03 | 9451 | 4079 |
| 2015/04 | 10382 | 5730 |
| 2015/05 | 12754 | 6354 |
| 2015/06 | 16718 | 8308 |
| 2015/07 | 13052 | 7338 |
| 2015/08 | 13121 | 12085 |
| 2015/09 | 15584 | 12736 |
| 2015/10 | 25107 | 11384 |
| 2015/11 | 60090 | 11402 |
| 2015/12 | 83500 | 16225 |
