# Supplementary material for: Forecasting electric vehicles sales with univariate and multivariate time series models: The case of China
Source: PLoS One. 2017 May 1;12(5):e0176729. doi: 10.1371/journal.pone.0176729 (PMC5411096; doi:10.1371/journal.pone.0176729)
Supplement: S2 Table — (DOCX) [file pone.0176729.s002.docx]

**S2 Table. Economic indicators from January 2011 to December 2015**

| Year/Month | Consumer price index | Consumer confidence index | Producer price index | Fuel retail price | Vehicle price | Baidu data |
| --- | --- | --- | --- | --- | --- | --- |
| 2011/01 | 104.9 | 99.9 | 106.6 | 100.2 | 101.28 | 1.661 |
| 2011/02 | 104.9 | 99.6 | 107.2 | 100.8 | 102.01 | 2.208 |
| 2011/03 | 105.4 | 107.6 | 107.3 | 99.7 | 101.89 | 2.592 |
| 2011/04 | 105.3 | 106.6 | 106.8 | 103.2 | 101.7 | 4.502 |
| 2011/05 | 105.5 | 105.8 | 106.8 | 101 | 101.63 | 2.484 |
| 2011/06 | 106.4 | 108.1 | 107.1 | 99.3 | 101.51 | 2.164 |
| 2011/07 | 106.5 | 105.6 | 107.5 | 99.8 | 101.88 | 1.962 |
| 2011/08 | 106.2 | 105 | 107.3 | 100.1 | 102.1 | 2.059 |
| 2011/09 | 106.1 | 103.4 | 106.5 | 100.3 | 102.08 | 2.323 |
| 2011/10 | 105.5 | 100.5 | 105 | 99.1 | 102.04 | 2.692 |
| 2011/11 | 104.2 | 97 | 102.7 | 99.6 | 102.01 | 2.58 |
| 2011/12 | 104.1 | 100.5 | 101.7 | 100.2 | 101.92 | 2.175 |
| 2012/01 | 104.55 | 103.9 | 100.73 | 100.1 | 99.82 | 2.043 |
| 2012/02 | 103.16 | 105 | 100.03 | 101.5 | 99.49 | 2.529 |
| 2012/03 | 103.59 | 100 | 99.68 | 103.1 | 99.18 | 2.641 |
| 2012/04 | 103.31 | 103 | 99.02 | 102.1 | 99.12 | 2.93 |
| 2012/05 | 103 | 104.2 | 98.6 | 98 | 99.35 | 2.623 |
| 2012/06 | 102.15 | 99.3 | 97.92 | 96.2 | 99.54 | 2.478 |
| 2012/07 | 101.78 | 98.2 | 97.13 | 97.5 | 99.62 | 2.237 |
| 2012/08 | 102.05 | 99.4 | 95.52 | 101.5 | 99.5 | 2.374 |
| 2012/09 | 101.91 | 100.8 | 96.45 | 102.8 | 99.72 | 2.856 |
| 2012/10 | 101.72 | 106.1 | 97.24 | 101.1 | 99.71 | 2.924 |
| 2012/11 | 102.04 | 105.1 | 97.08 | 99.7 | 99.54 | 2.851 |
| 2012/12 | 102.52 | 103.7 | 98.06 | 99.5 | 99.36 | 2.754 |
| 2013/01 | 102.03 | 104.5 | 98.36 | 100.2 | 99.8 | 3.195 |
| 2013/02 | 103.22 | 108.2 | 98.37 | 100.2 | 100 | 2.283 |
| 2013/03 | 102.07 | 102.6 | 98.8 | 101 | 100.01 | 3.289 |
| 2013/04 | 102.39 | 103.7 | 97.38 | 98.1 | 100.05 | 2.395 |
| 2013/05 | 102.1 | 99 | 97.13 | 98.4 | 100.21 | 2.184 |
| 2013/06 | 102.67 | 97 | 97.3 | 99.6 | 100.5 | 2.109 |
| 2013/07 | 102.67 | 97.2 | 97.73 | 100.4 | 100.53 | 3.384 |
| 2013/08 | 102.57 | 97.8 | 98.37 | 101 | 100.64 | 3.203 |
| 2013/09 | 103.05 | 99.8 | 98.66 | 101.6 | 100.6 | 3.109 |
| 2013/10 | 103.21 | 102.9 | 98.49 | 99.3 | 100.73 | 3.17 |
| 2013/11 | 103.02 | 98.9 | 98.58 | 99.6 | 101.1 | 3.08 |
| 2013/12 | 102.5 | 102.3 | 98.64 | 100.7 | 100.9 | 3.12 |
| 2014/01 | 102.49 | 101.1 | 98.36 | 100.1 | 100 | 2.633 |
| 2014/02 | 101.95 | 103.1 | 97.98 | 99.3 | 99.9 | 4.694 |
| 2014/03 | 102.38 | 107.9 | 97.7 | 100.7 | 99.5 | 4.856 |
| 2014/04 | 101.8 | 104.8 | 98 | 99.4 | 99.2 | 4.116 |
| 2014/05 | 102.48 | 102.3 | 98.55 | 100.3 | 98.8 | 4.24 |
| 2014/06 | 102.34 | 104.7 | 98.89 | 100 | 98 | 4.874 |
| 2014/07 | 102.29 | 104.4 | 99.13 | 100.1 | 97.7 | 5.026 |
| 2014/08 | 101.99 | 103.8 | 98.8 | 98.6 | 96.9 | 5.964 |
| 2014/09 | 101.63 | 105.4 | 98.2 | 98.6 | 96.3 | 6.429 |
| 2014/10 | 101.6 | 103.4 | 97.76 | 98.4 | 96.1 | 6.756 |
| 2014/11 | 101.44 | 105.5 | 97.31 | 97 | 95.9 | 6.971 |
| 2014/12 | 101.51 | 105.8 | 96.68 | 97.5 | 95.5 | 7.29 |
| 2015/01 | 100.76 | 105.7 | 95.68 | 95.6 | 100.2 | 7.82 |
| 2015/02 | 101.43 | 109.8 | 95.2 | 98.9 | 100.1 | 5.96 |
| 2015/03 | 101.38 | 107.1 | 95.44 | 102.5 | 99.5 | 7.51 |
| 2015/04 | 101.51 | 107.6 | 95.43 | 99.3 | 98.5 | 7.23 |
| 2015/05 | 101.23 | 109.9 | 95.39 | 102.3 | 97.1 | 7.19 |
| 2015/06 | 101.39 | 105.5 | 95.19 | 99.7 | 95.7 | 6.802 |
| 2015/07 | 101.65 | 104.5 | 94.63 | 98.5 | 95.6 | 6.362 |
| 2015/08 | 101.96 | 104 | 94.8 | 96.9 | 95.1 | 6.93 |
| 2015/09 | 101.6 | 105.6 | 94.5 | 98.4 | 94.8 | 7.389 |
| 2015/10 | 101.27 | 103.8 | 94.1 | 100.3 | 95.2 | 8.653 |
| 2015/11 | 101.49 | 104.1 | 94.1 | 99.4 | 96.1 | 8.993 |
| 2015/12 | 101.64 | 103.7 | 94.1 | 98.9 | 96.8 | 9.958 |
